# Why Not Just Features? Reconsidering Infants’ Behavior in Individuation Tasks

**DOI:** 10.3389/fpsyg.2020.564807

**Published:** 2020-10-21

**Authors:** Frauke Hildebrandt, Jan Lonnemann, Ramiro Glauer

**Affiliations:** ^1^Department of Social and Educational Science, University of Applied Sciences Potsdam, Potsdam, Germany; ^2^Empirical Childhood Research, University of Potsdam, Potsdam, Germany

**Keywords:** object individuation, object index, individuation failures, feature-based learning, spatiotemporal individuation, frame of reference

## Abstract

It counts as empirically proven that infants can individuate objects. Object individuation is assumed to be fundamental in the development of infants’ ontology within the object-first account. It crucially relies on an object-file (OF) system, representing both spatiotemporal (“where”) and categorical (“what”) information about objects as solid, cohesive bodies moving continuously in space and time. However, infants’ performance in tasks requiring them to use featural information to detect individuation violations appears to be at odds with the object-first account. In such cases, infants do not appear to be able to develop correct expectations about the numerosity of objects. Recently, proponents of the object-first account proposed that these individuation failures result from integration errors between the OF system and an additional physical reasoning system. We are going to argue that the predictions of a feature-based physical-reasoning (PR) system are sufficient for explaining infants’ behavior. The striking predictive power of the PR system calls into question the relevance of the OF system and, thereby, challenges the assumption that infants can individuate objects early on.

## Objects First

The results of a large number of empirical studies are regarded as evidence that infants can individuate objects within the first year of life. The standard interpretation of the results is known as the object-first hypothesis (Xu and Carey, 1996). According to this hypothesis, children organize information in their visual field much like adults do, namely, in terms of space, object, and movement (Xu et al., 2004; Xu, 2007). Objects are seen as cohesive entities that move continuously through space and time (Carey, 2009; Cacchione and Rakoczy, 2017). Spatiotemporal information about location and motion is seen as the primary information adults use to individuate objects (Kahneman et al., 1992; Pylyshyn, 2001). Moreover, it is also seen as the basis on which infants learn to individuate objects – the very core of their ontology (Moore et al., 1978; Wynn, 1992; Spelke et al., 1995; Xu and Carey, 1996; Hespos and Rochat, 1997; Wilcox and Baillargeon, 1998; Aguiar and Baillargeon, 1999; Van de Walle et al., 2000; Santos et al., 2002; Xu and Baker, 2005; Mendes et al., 2008; Yoon et al., 2008; Futó et al., 2010; among others).

It is further assumed that infants use featural information to individuate objects from around 12 months of age. Only then they appear to individuate two objects in occlusion events by feature differences without having seen both objects synchronously (Xu and Carey, 1996; Van de Walle et al., 2000; Xu and Baker, 2005). Similar abilities have also been found in nonhuman primates (Santos et al., 2002; Phillips and Santos, 2007; Mendes et al., 2008, 2011). According to the object-first hypothesis, it seems to be demanding for children to handle the interplay of spatiotemporal information processing and featural object-identification (e.g., Leslie et al., 1998; Wilcox and Baillargeon, 1998; Krøjgaard, 2000; Bonatti et al., 2002; Wilcox and Chapa, 2002; Rivera and Zawaydeh, 2007; Futó et al., 2010; Surian and Caldi, 2010).

Stavans et al. (2019) attempted to reconcile these findings with research on infants’ abilities of physical reasoning. The latter suggests that infants are able to use featural information to guide their understanding of physical events much earlier than for object individuation. It was proposed that object individuation relies on the interplay of two cognitive systems, an object-file system (OF system) and a physical-reasoning system (PR system). Under certain conditions, these systems produce contradictory predictions about how many objects are involved in an event, leading to no expectations at all – so-called “catastrophic individuation failures.” We are going to discuss each system in turn before arguing that object individuation is cognitively more demanding than suggested by the object-first account. Moreover, the introduction of a PR system draws into doubt whether an OF system is necessary for producing the observed expectations in infants. Thereby, it becomes doubtful whether infants individuate objects.

## Object-File System

The ability to individuate objects is assumed to centrally rely on an object-file system (OF system), which allows children to integrate spatiotemporal (“where”) information and categorical (“what”) information about objects. There are different object-file accounts: either object-representation information is stored in an object’s file, and spatiotemporal information is used to pick out this file (Kahneman et al., 1992; Gordon and Irwin, 1996), or an index is seen as fixed to an object and remaining there as it moves and object-representation information can be fixed to the index (Pylyshyn, 1989, 2009; Leslie et al., 1998). Stavans et al. (2019), however, interpreted both processes as separate but closely related mechanisms within the OF-System. Overall, object files were invoked to explain the ability to represent objects and their features. Adults can identify (and misidentify) objects because they have a mental representation of objects that binds together different strands of information about an object. Note that the OF system is, among other things, intended to explain how participants represent objects as cohesive entities that remain the same over time, that is, it is intended to explain spatiotemporal individuation.

Butterfill (2020, p. 58) illustrates the OF system with the help of an analogy: consider a logistician who takes track of her company’s trucks with the help of pins on a map. “For each pin, there is a corresponding truck and, ideally, the movements of the pin reflect the movements of the truck it corresponds to. […] Object indexes are a mental counterpart of the pins: they are things that point to, or index, objects” (ibid.). Additionally, the OF system is said to use certain kinds of categorical information to create object representations. It is assumed that at the beginning, only basic-level ontological categories are distinguished (human vs. non-human and animate vs. inanimate). Only later, ordinary object categories like “ball,” “duck,” or “puppet” are distinguished. Which kind of categorical information is available to the OF system changes over development.

## Physical-Reasoning System

Based on the corpus of relevant studies, Stavans et al. (2019) concluded that infants at 12 months of age and younger do not always recognize individuation violations, that is, they do not expect the correct number of objects presented at the end of an occlusion event. Individuation becomes especially difficult for infants when two different objects from the same basic-level category are used, e.g., two balls that differ in size, pattern, and color. To find an explanation for these findings, Stavans et al. (2019) postulated a second system, the physical-reasoning (PR) system.

The physical-reasoning system is a causal-reasoning system that predicts object interactions over time (Baillargeon et al., 2009, 2012; Mascalzoni et al., 2013; Wang and Goldman, 2016). It allows to classify types of events (e.g., occlusion and containment) and features of the involved objects that have already been identified as causally relevant for certain types of events (e.g., inert or self-propelled, open or closed surfaces, and arrangement; Baillargeon et al., 2009). The PR system was presented as using the information provided by the OF system in order to structure an observed event and to ascribe different roles to objects involved in the event, such as occluder and occludee in occlusion events.

According to Stavans et al. (2019, p. 197), object individuation has to be seen as the “part and parcel of infants’ ability to represent and reason about physical events.” Stavans et al. (2019) stated that it depends on implicit knowledge infants already acquired about event-specific causally relevant object features. The PR system uses “whatever featural information has been identified as causally relevant for the event category involved” (ibid., p. 219). Object representations are only said to provide the PR system with additional featural information that it needs in order to produce expectations about the course of an event.

## The Role of the *OF* and *PR* Systems in Producing Infants’ Expectations

Stavans et al. (2019) have grouped object-individuation experiments into six experimental paradigms as summarized in Table 1 (see Stavans et al., 2019, p. 206). Table 1 represents infants’ expectations in relevant experiments (including incorrect expectations) and sets them against the corresponding predictions of the OF and PR systems. The OF system is thought to produce predictions based on categorical or spatiotemporal information, while the PR system uses featural information that is known to be relevant in a given event. In case B, for instance, infants observe how two different objects of the same category (say, a small blue ball and a bigger red ball) emerge from behind an occluder and disappear again behind it. Both objects are visible at the same time. Because they are located at different places (left and right of the occluder), the OF system is thought to use this spatiotemporal information to register two objects. Because the objects differ featurally, the PR system likewise registers two objects. In case D, however, these same-category objects are not simultaneously visible as they emerge from and disappear again behind the occluder subsequently. The OF system, having access neither to categorical nor to spatiotemporal information that would distinguish between the two balls, registers only one object. The PR system is still able to use featural information to predict two objects. When the predictions of both systems agree, as in experimental settings A, B, and C, infants’ expectations are simply the result of these predictions. But when these predictions disagree quantitatively, as in case D, a conflict ensues. As a result, no expectations are formed about how many objects there are behind the occluder.

**Table 1:** Infants’ performance in different individuation tasks according to the new model of early individuation (Stavans et al., 2019, p. 206).

Event		Task	Object-file (OF) number of hidden objects	Physical-reasoning (PR) number of hidden objects	OF and PR agree?	Infants’ expectation
Same-Object	A	Standard and search (Wilcox and Baillargeon, 1998; Van de Walle et al., 2000; Wilcox and Chapa, 2002; Wilcox, 2007; Stavans et al., 2019)	1	1	Agree	1
Different-Objects	B	Different-locations standard and search (Xu and Carey, 1996; Van de Walle et al., 2000; Xu et al., 2004; Zosh and Feigenson, 2015)	2	2	Agree	2
	C	Different-categories standard and search (Bonatti et al., 2002, 2005; Surian and Caldi, 2010; Setoh et al., 2013)	2	2	Agree	2
	D	Same-category standard and search (Xu and Carey, 1996; Wilcox and Baillargeon, 1998; Van de Walle et al., 2000; Bonatti et al., 2002; Wilcox and Chapa, 2002; Xu et al., 2004; Surian and Caldi, 2010)	1	2	Disagree quantitatively	Catastrophic failure: no expectation
	E	Same-category one-event (Wilcox and Chapa, 2002)	1	2	Not applicable: ongoing event, PR has priority	2
	F	Same-category remainder (Wilcox and Baillargeon, 1998; Wilcox and Schweinle, 2002; Xu and Baker, 2005; McCurry et al., 2009)	0	1	Disagree qualitatively	1

Each row represents a different individuation task (see Stavans et al., 2019 for a detailed description of the different tasks) as well as the numbers of hidden objects expected by the OF or the PR system in the respective task, whether these expectations match or not, and the empirically observed expectations of infants. We have included references to the publications in which the respective experiments are described.

Table 1 shows the following:

1. In five out of six cases (A–C, E–F), the expectation of children can be predicted by the PR system.
2. In three out of six cases (D–F), the OF system and PR system provide different predictions. In two of these three cases (E and F) the predictions of the PR system match infants’ behavior, those of the OF system do not match. That is, the PR system overrides the OF system’s predictions.
3. In the sole case in which the OF system and PR system provide different predictions and the OF prediction is not overridden by the PR system (D), both predictions do not correspond to infants’ behavior.

This means that the PR system alone predicts infants’ expectations in five out of six cases. In three out of six cases, the assumption of an OF system leads to false predictions of infants’ expectations. In another three out of six cases, the assumption of an OF system leads to the same predictions as the assumption of a PR system. Moreover, in the single case where the PR system does not predict the empirically observed expectations, neither does the OF system. This alone casts doubt on the view that the OF system plays a role in generating expectations about the respective events at all. The explanatory burden can be carried by the PR system alone. Nonetheless, Stavans et al. (2019) assume *two* systems. A possible reason is that Stavans et al. (2019) underestimate the potential of a purely feature-based cognitive mechanism.

## Feature-Based *PR* System Without Object Individuation

The PR system is presented by Stavans et al. (2019) as processing categorical information provided by the OF system. For the PR system, however, it is irrelevant whether this information is bound to object representations. Instead of interpreting such information as pertaining to all exemplary of a kind, it can be seen as featural information about a familiar feature pattern. Correspondingly, it does not seem necessary to assume that the processing of such information requires a reference to objects. The PR system can arguably fulfill its function without relying on object representations. We thus suggest that a PR system might well function without recourse to an OF system. All that is required is understanding features somewhat more broadly.

Following Cohen et al. (2002), we would like to suggest to use “feature” in the following way: features correspond to perceptual differences. Each modality (vision, audition, touch, olfaction, etc.) provides access to different features. Furthermore, features combine to form complex features/feature patterns, and feature changes and interactions. Note that we make no claim about how features are neuro-physiologically or functionally integrated in the CNS.

Features are holistic in that they are (always) part of a perceptual scene, and they are dynamic in that perception is a temporally extended process. Features, feature patterns, and feature changes can be compared and accessed by their similarities and differences. Perceived feature correlations and interactions can be generalized and used to predict outcomes in new perceptual situations. Basic feature interactions are what is perceived when confronted with what, for human adults, are events like occlusions (e.g., Xu and Carey, 1996), collisions (e.g., Baillargeon, 1986), or containments (e.g., Van de Walle et al., 2000) as also operationalized in many standard experiments (see Stavans et al., 2019). Perceiving feature interactions differs from perceiving events in that the latter involves object individuation and the former does not: events contain objects.

As a result, some feature patterns might correspond to what is regularly called categorical information. Take a duck. The (multimodal and dynamic) feature pattern that is perceived when confronted with a duck can be distinguished from (multimodal) feature patterns that are perceived when facing a dog as well as from feature patterns that result from facing a lake and its surrounding reeds. Findings from Kingo and Krøjgaard (2011) show that the modality in which features are accessed clearly influences how behavior is attuned to what human adults perceive as particular objects. Similarly, some feature patterns or feature changes might correspond to what, in this context, is called spatiotemporal information. What we interpret as the movement of a duck could be perceived as a relative feature change within a broader feature pattern. For a comparably low-level interpretation of object individuation experiments see Krøjgaard et al. (2013).

Cohen et al. (2002) provided an information processing account of perceptual development that can explain how stable representations of correlated features emerge. Representations of high-level feature patterns are built up by hierarchically combining simpler feature representations. Features can be conceived as discriminable properties of the sensory impression. Expectations about future observations are formed on the basis of these feature patterns and familiar regularities of feature changes. Vierck and Miller (2005) have empirically demonstrated that featural information can serve as a source for the construction of expectations.

We propose that infants build expectations based on such a feature model. In particular, there may be no numerical expectations in infants, only expectations concerning composite feature patterns. For instance, a classical experiment that is taken to show that infants individuate objects based on spatiotemporal information can then be re-interpreted as follows: in a study with two occluders made by Xu and Carey (1996), objects are chosen such that they cannot be distinguished based on their features alone. In one version, two yellow ducks subsequently appear and disappear again from behind two different occluders. The occluders are then lifted. Infants show surprise when only one duck is behind one of the occluders and there is no duck behind the other. It is argued that infants understand that there should be two ducks because the occluders are separated, and if there were only one object it would have had to pass the space between the occluders – after all, the two ducks could not be distinguished based on their features alone (Spelke and Kestenbaum, 1986; Xu and Carey, 1996). That the ducks are not featurally different, however, does not imply that there is no featural information available in the overall input that could be used to distinguish between what adults would describe as one duck appearing from behind an occluder and disappearing again and two ducks subsequently appearing from and disappearing behind two different occluders. For instance, it is possible to distinguish one-occluder events from two-occluder events based on the edges (that is, discontinuities of image brightness) that correspond to the borders between the occluders and the background.

This information could then be used to form expectations about subsequent observations. It suffices to use featural information in order to distinguish between the two cases. Consider, for example, a video recording of a scene and the information it can capture without actually counting objects. Cameras merely capture featural information of a scene and store them for later use. As a medium, they do not encode objects *per se*, just patches of color that are structured in a way that allows us to see them as objects. Thereby, the stored featural information can be used as information about objects. But strictly speaking, video displays create the illusion of movement where nothing moves. Only pixels change their color in an orderly way.

Note that there is always some sort of featural information available that could be used to distinguish events according to the numerosity of the involved objects: all perceptible numerical differences have corresponding featural differences. A visual array with two objects in it provides a different feature pattern impression than an array with only one object. The featural information alone can suffice to form expectations about features and feature changes without retorting to representations of individual objects.

Artificial systems can serve as a proof of concept that information pertaining to different feature patterns can, in principle, be processed without additional, OF-like representations of individual objects. Consider, as an illustration, so-called object recognition in AI. A now-standard way of extracting which kinds of “objects” are visible in a scene uses convoluted neural networks (CNNs) that extract low-level features from overlapping feature-sections of an image. The nodes of a network, such as a convolution layer, are sensitive to certain feature parts of the image. These features are then further processed to obtain more complex features (or feature patterns) and, eventually, to give the category of “objects” that are visible (cf., e.g., Krizhevsky et al., 2012). When the output layer is structured accordingly, such systems can be used to detect and categorize several “objects” at different “positions” in an image or video recording (cf., e.g., Redmon and Farhadi, 2017). Such detections are based on featural information alone. The process does not require object representations as postulated for the OF system. Notably, it could be demonstrated that neural networks (Schlesinger, 2003), as well as a physical humanoid robotic system (Lovett and Scassellati, 2004), produce eye-movement/camera-movement outputs that are comparable to Baillargeon’s (1986) findings in infants.

Also, featural information can be used to create expectations about how a featural scene will unfold. For instance, predictive coding strategies can be used to build recurrent CNNs that predict subsequent frames of a video (cf., Lotter et al., 2016). Such predictions function without representations of objects and break down when the presented video sequence lacks cohesion, such as when the order of video frames is randomly scrambled.

Note that we are not claiming that the infant cognitive system processes information in the same way or significantly similar to these AI systems. The artificial systems are to show that featural information can in principle be processed in a way that corresponds to observed infant behavior. Moreover, we assume that the adult cognitive system does not process information in the same way as such AI systems – for reasons formulated in Hildebrandt et al. (in press). This is in line with Geirhos et al. (2018) who have shown that there are clear performance differences in object recognition tasks between deep learning neural networks and adult human participants, especially when images are distorted. This is interpreted as evidence that, as of now, neural networks that are used for object recognition do not provide an explanation of how human adults recognize what kind of object is presented. In our terminology, this is because neural networks process only feature patterns but human adults individuate objects.

We are now in the position to explain infants’ behavior in case D (see Table 1) within the feature-based PR system: infants build up expectations based on already experienced regularities of certain feature-interactions – such as occlusions, containments, or collisions. Infants who have already acquired feature-based physical knowledge expect interactions of certain features, such as shape, size, pattern, color, or function, and draw conclusions about which feature changes are likely to occur in which interactions and which are not. Thus, expectations not only depend on the ability to detect certain kinds of features, but they also depend on having acquired knowledge about certain kinds of *feature interactions*: while a particular feature might be considered relevant for predictions in a specific feature interaction, the same feature might not be considered as relevant in other feature interactions. Without this interaction-specific knowledge, infants lack the basis for specific expectations.

Consider again the individuation tasks of case D described above, involving, for instance, two different balls. When both balls are simultaneously presented, infants form an adequate expectation and are surprised if only one ball appears when the occluder is lifted. When the two balls are presented subsequently, infants do not show surprise when they are presented with only one ball. From this, it is concluded that infants can use alleged spatiotemporal information (their relative position) for individuating objects but not featural information (their different sizes and colors). We can now see that both kinds of information can be interpreted as different feature patterns. The complex feature pattern consisting of the simultaneous presentation of small-blue-ball-shape and bigger-red-ball-shape leads to the expectation of an equivalent pattern when the occluder is lowered. The subsequent presentation of the small-blue-ball-shape and bigger-red-ball-shape, however, is more complicated. Expectations cannot simply be based on the observed feature pattern. After all, there was a featural difference between the first and the second presentations. Therefore, infants’ expectations could neither be a “copy” of the first nor of the second feature pattern. Correspondingly, infants are not led to expect either of the simpler patterns. Moreover, as long as infants are unfamiliar with the specific kind of feature change, no expectations of a complex small-blue-ball-shape-and-bigger-red-ball-shape pattern could be formed. As a result, infants do not expect a feature pattern that would correspond to either what adults see as one object or what adults see as two objects.

We propose that a feature-based approach might as well be used to explain findings such as the ones from Stavans et al. (2019), findings from tasks involving verbal communication (Yoon et al., 2008), findings from manual search tasks (Van de Walle et al., 2000), and findings concerning object working memory capacity in infancy (Kibbe and Leslie, 2013). In all of these cases, a featural difference can be found that can serve to explain performance differences in the experimental conditions.

As a result, reasoning about physical events can be interpreted as reasoning about feature interactions and feature changes. This implies that infants need not have a grasp of spatiotemporal identity (Strawson, 1959; Quine, 1960), as postulated by the object-first interpretation, in order to solve so-called object individuation tasks. Moreover, there are additional considerations against the idea that an OF system could ground object individuation, to which we will now turn.

## Critique of the *OF* System

From a common-sense perspective, it might seem counterintuitive that young children lack the ability to individuate objects while displaying the ability to classify based on feature similarities. The view that object individuation precedes classification abilities is central in classical accounts of linguistic meaning according to which this meaning consists in name-object assignments (Mill, 1843; Augustine, 2006; Lycan, 2008). But this was sharply criticized in twentieth-century analytic philosophy. Wittgenstein (1969), for instance, pointed out that many of the words we use simply do not refer to spatiotemporal objects or events and, thereby, shows that the referential theory of linguistic meaning is inadequate. Chomsky later argued that even proper names, for which the word-object model would be most plausible, do not function as word-object-assignments (Chomsky, 2000; Sheehan and Hinzen, 2011).

The major philosophical topic in this debate was how reference to objects is possible at all (cf., e.g., Frege, 1892; Russell, 1905; Searle, 1958; Strawson, 1959; Quine, 1960; Donnellan, 1966; Tugendhat, 2016; Kripke, 1980; Evans, 1982). Answering this question is arguably problematic because it requires being able to explain how we segregate the environment into objects. Hirsch (1997) lays out the underlying difficulties by showing that there is an indefinite number of possible interpretations of what is going on in the world: “there is the hypothesis that babies are Humeans who believe only in momentary events and their interrelations (Hume, 1739); or that they are Butlerians who only believe in mere logically intact substances that cannot survive the loss of any parts (Butler, 1897); or that they are Strawsonian feature-placers” (Strawson, 1959; Hirsch, 1997, p. 410). Because there are so many ways of organizing the sensory input, when it comes to cognitive development, we need to explain how we come to structure the world in our particular way. That is, we need to explain how we come to segregate our sensory input into objects with spatiotemporal identity criteria in the first place. We cannot simply assume that the world falls into objects and think that all that needs to be explained is how we relate to the objects in our environment. In other words, the explanatory problem is not how objects are assigned to our mental representations, it is how the sensory input is processed in this particular way.

This is also why object files and object indexes cannot explain object individuation. A theory of object individuation, which Pylyshyn (1989, 2009), as well as Leslie et al. (1998), explicitly set out to formulate, would have to explain how a sensory input which is not yet structured into objects is processed in a way that allows object individuation. The explanation would have to supply individuation criteria for objects. In our common understanding, objects are individuated by the spacetime region they occupy during their existence. One object cannot occupy different places at the same time, and all places it occupies over time are connected by a continuous trajectory. Thus, individuation criteria for (physical) objects are spatiotemporal. That is, objects are individuated by their place at different moments. This notion of an object is precisely what Leslie et al. (1998, p. 11) attempt to capture and which the object-first interpretation relies on. But neither object indexes nor object files provide spatiotemporal individuation criteria.

As places are either used to address a file or to fix an index, using an object file or an index in order to “bind” featural information and, thus, to create a representation of an object, presupposes a spatiotemporal frame of reference.

To illustrate: object indexing is sometimes compared to pointing to an object. Pointing, however, can only serve to pick out an object if the communication partner already knows that it is objects we are pointing to. Only then, pointing can serve to single out one object among others. For feature-placers, say, pointing would serve to highlight a feature section. Pointing cannot by itself serve to convey the idea of an object (or a place) – just as it cannot convey the idea of a point in time. The OF system, in effect, presupposes the spatiotemporal individuation of objects, and is, therefore, unable to explain how objects are individuated as one and the same in the first place.

In effect, object individuation is directly presupposed in Butterfill’s (2020) illustration of indexing. Butterfill compared indexing with assigning a pin on a map to the different trucks of a truck company in order to keep track of their current position. According to this picture, individual objects are tracked by having assigned a particular index, just as each truck gets assigned a particular pin. Note that the individuation of objects thereby depends on (i) that indexes are already individuated as one and the same at a certain moment and (ii) that assigning an index to an object presupposes that objects are likewise individuated as one and the same.

Overall, the debate neglects and Stavans et al. (2019) overlooked, that object files and indices cannot serve to ground object individuation. Explaining object individuation would require a recurrence to how access to spatiotemporal identity criteria (that is, places and times) is developed.

These theoretical considerations suggest that object individuation is cognitively demanding. This is also supported by empirical findings suggesting that object individuation in adults requires selective attention (Burr et al., 2010) and competes for cognitive resources with working memory during tasks involving both simultaneous enumeration (Piazza et al., 2011) as well as sequential enumeration (Cheng et al., 2019). Together with reasons of parsimony, this suggests that infants’ behavior in individuation tasks should not be regarded as evidence for their ability to individuate objects. As a result, what has been proposed as a comprehensive explanation of the range of experimental findings by Stavans et al. (2019), should rather open the debate to reconsider the cognitive basis of infants’ performance in the experiments listed above.

## Outlook and Conclusion

We interpret the presented findings on object individuation as follows and make the following predictions as well as suggestions:

1. That infants fail in case D shows that they cannot individuate objects. Otherwise, they would reliably infer the number of objects involved from feature differences – regardless of the features that are involved. Objects have spatiotemporal individuation criteria. If infants were using spatiotemporal individuation criteria, they would not systematically fail to form correct expectations.
2. That infants do not fail in case A does not show that they reason from sameness to number (identity) – that is from feature to quantity. That infants do not fail in the classical task from Xu and Carey (1996), in cases B and C, and later in case D does not show that they are able to individuate objects. Instead of relying on spatiotemporal information, each of these tasks can be solved by using information about the specific changes features (and larger feature patterns) undergo in various familiar feature interactions (what we call events). With increasing familiarity, young children can make ever more accurate predictions about the course of feature changes they are presented with. The pattern of “successes” in individuation tasks is thus revealing as to which features infants find relevant in certain feature interactions. In our account, it is, for one, likely that infants are not able to individuate objects, and for another, the listed experiments are unsuitable to provide evidence for object individuation. They provide interesting evidence about which kinds of features are processed by infants when confronted with certain feature interactions (e.g., occlusions, containments, and collisions) and how this develops.
3. Arguably, different experimental paradigms are needed if we are to test for children’s capacity to individuate objects. As noted, objects have spatiotemporal individuation criteria. If we are to distinguish object individuation from feature processing, we need to find tasks in which spatiotemporal identity criteria are used independently of features. An experimental paradigm that is apt for testing object individuation abilities would thus have to exclude the possibility that the tasks are solved based on feature processing alone. For example, a paradigm to assess the sensitivity to errors of misidentification could be applied (see Hildebrandt et al., in press). Such errors of misidentification threaten to occur when there are several featurally indistinguishable objects only one of which is “the right one.” Which object is “the right one” could depend on various properties such as a hidden function (only one is magnetic or has a functioning light) or a particular history (the one we have played with before or the one that was given to a child). Importantly, the distinguishing property would have to be one that is motivating for test participants and could not be tracked during test based on features, including the overall feature pattern. The test situation would have to be such that the featurally indistinguishable objects could easily be confused. For instance, there could be several toy fire engines of the same make only one of which has a working siren. The procedure could consist of a familiarization phase during which child and experimenter play with these toys, making sure that the child realizes that only one has a working siren. The toys are then stored away ensuring that the toy with the working siren cannot be tracked. This could, for instance, be achieved by placing them in an intransparent box that is then shaken or spun. Participants could then be offered to pick one of the toys. The test phase would undergo a similar procedure. When the toys are to be stored away, children’s sensitivity to the threat of confusing the toys could be measured. For instance, children could be offered two boxes to store away the toys, one containing compartments that would keep the toys apart when the box is shaken. Children that are sensitive to errors of misidentification should be more likely to choose the compartmentalized box.

In summary, the main goal of this article is to shed doubt on the object-first account of cognitive development by showing that, under the assumption that infants individuate objects, it is difficult to explain infant performance in object individuation tasks. The explanation proposed by Stavans et al. (2019) has to invoke two cognitive systems and makes *post-hoc* assumptions about their interaction in several cases. There is a simpler alternative explanation available that is very much in line with how Stavans et al. (2019) present their physical-reasoning system and which does not require making additional assumptions for each case. Instead of attributing object representations and then having to explain why they collapse under various conditions, this explanation proposes that infants process feature patterns and that the adequacy of their expectations primarily depends on their familiarity with certain feature interactions. Additional theoretical considerations were presented to the effect that object individuation is cognitively demanding and depends on the acquisition of spatiotemporal identity criteria.

## Author Contributions

FH conceived of the original idea. JL initiated reinterpreting all discussed experiments in detail. RG introduced examples from AI. FH and RG elaborated the argument. All authors contributed to the article and approved the submitted version.

### Conflict of Interest

The authors declare that the research was conducted in the absence of any commercial or financial relationships that could be construed as a potential conflict of interest.
